# Phloroglucinols with Antioxidant Activities Isolated from *Lysidice rhodostegia*

**DOI:** 10.3390/molecules22060855

**Published:** 2017-05-23

**Authors:** Xian-Fu Wu, Li Li, Yong Li, Hai-Ning Lv, Yun-Bao Liu, You-Cai Hu

**Affiliations:** 1 State Key Laboratory of Bioactive Substance and Function of Natural Medicines, Institute of Materia Medica, Chinese Academy of Medical Sciences and Peking Union Medical College, Beijing 100050, China; annaleelin@imm.ac.cn (L.L.); liyong@imm.ac.cn (Y.L.); lhn371102@126.com (H.-N.L.); liuyunbao@imm.ac.cn (Y.-B.L.); 2 National Institutes for Food and Drug Control, Beijing 100050, China

**Keywords:** phloroglucinol, *Lysidice rhodostegia*, electronic circular dichroism (ECD) calculation, antioxidative activity

## Abstract

Two new phloroglucinols, lysidisides X and Y (**1** and **2**), and two known compounds, 2-(2-methylbutyryl)phloroglucinol 1-*O*-β-d-glucopyranoside (**3**) and (*E*)-resveratrol 3-(6″-galloyl)-*O*-β-d-glucopyranoside (**4**), have been isolated from the roots of *Lysidice rhodostegia*. The structures of **1** and **2** were elucidated primarily by NMR experiments. Their absolute configurations were deduced via circular dichroism (CD) data and electronic circular dichroism (ECD) calculations. Compounds **1** and **2** exhibited significant antioxidative activities with IC_50_ values of 12.0 and 11.8 µM, respectively.

## 1. Introduction

*Lysidice* (Leguminosae) are shrubs or trees with two species widely distributed in the south and southwest of China, and in Vietnam*. Lysidice rhodostegia* is a medicinal plant, and its roots are mildly toxic and have been used in Chinese folk medicine for the treatment of ache, fracture, and hemorrhage [1]. As part of a program to search for new bioactive natural products from poisonous plants, we investigated the roots of *L. rhodostegia*. Our prior work on this plant afforded structurally diverse and biologically active metabolites, such as phloroglucinols, flavonoids, stilbenes, and triterpenoids, some of which displayed potent vasodilatory and antioxidative activities [2,3,4,5,6,7,8,9]. During an ongoing search for new bioactive natural products from this plant, two new phloroglucinols, lysidisides X and Y (**1** and **2**), along with two known compounds, 2-(2-methylbutyryl)phloroglucinol 1-*O*-β-d-glucopyranoside (**3**) [10] and (*E*)-resveratrol 3-(6″-galloyl)-*O*-β-d-glucopyranoside (**4**) [11], were isolated from its roots. Especially, naturally-occurring 4-arylflavan-3-ols are relatively rare and only three examples have been reported: (2*R*,3*R*,4*S*)-4-(2,4,6-trihydroxyphenyl)flavan-3,3′,4′,5,7-pentaol from *Nelia meyeri* Schwant [12], and lysidisides V and W from *L. rhodostegia* in our previous work [9]. Compound **1** represents the first 4-arylflanvan-3-ol incorporating oxirane, and compound **2** is a new member of the phloroglucinol family. Herein, we report the isolation, structure elucidation, and antioxidative activity of **1** and **2**.

## 2. Results and Discussion

### 2.1. Purification of Compounds ***1***–***4***

**Figure 1:**
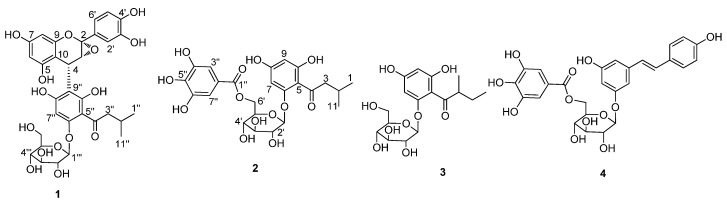
Structures of compounds **1**–**4**.

The dried roots of *L. rhodostegia* were powdered and extracted with 95% ethanol. The crude extract was suspended in water, and then successively extracted with *n*-hexane, EtOAc, and *n*-BuOH. The EtOAc extraction was subjected to repeated column chromatography on ODS (ostade-cylsilane), Sephadex LH-20, and preparative HPLC to provide four compounds: lysidiside X (**1**), lysidiside Y (**2**), 2-(2-methylbutyryl)phloroglucinol 1-*O*-β-d-glucopyranoside (**3**), and (*E*)-resveratrol 3-(6″-galloyl)-*O*-β-d-glucopyranoside (**4**) (Figure 1). Compounds **1** and **2** were evaluated for their antioxidative activity in vitro.

### 2.2. Structure Elucidation of Compounds ***1***–***4***

Lysidiside X (**1**) was obtained as pale yellow powder. It gave a pseudomolecular ion [M + Na]^+^ peak at *m*/*z* = 681.1763 (calcd. for C_32_H_34_O_15_Na, 681.1795) by HRESIMS, consistent with the molecular formula C_32_H_34_O_15_ and accounting for 16 degrees of unsaturation. The ^1^H- and ^13^C-NMR spectra (Table 1) showed the resonances for three oxygenated aromatic carbons (δ_C_ 161.2, 160.2, 159.6), three up-shielded aromatic carbons (δ_C_ 110.4, 108.2, 96.2), one conjugated carbonyl carbon (δ_C_ 208.2), one methine (δ_H_/δ_C_ 2.21/26.1), one methylene (δ_H_/δ_C_ 3.14, 2.88/54.3), and two methyls (δ_H_/δ_C_ 0.89/22.9, 0.92/23.3) (Supplementary Materials Figures S2 and S3). These data, together with those for glucosyl unit (δ_C_ 102.1, 78.4, 78.3, 74.9, 71.2, 62.3), suggested the presence of an isovalerylphloroglucinol glucoside moiety [2,6,9]. In addition, the ^1^H- and ^13^C-NMR spectra (Figures S2 and S3) displayed the signals of 1,2,3,5-tetrasubstituted aromatic ring [δ_H_ 6.03 (1H, d, *J* = 2.0 Hz) and 5.94 (1H, d, *J* = 2.0 Hz); δ_C_ 158.4, 156.8, 154.1, 103.7, 98.4, 96.6], 1,2,4-trisubstituted aromatic ring [δ_H_ 6.91 (1H, dd, *J* = 8.0, 2.0 Hz), 6.88 (1H, d, *J* = 2.0 Hz), and 6.56 (1H, d, *J* = 8.0 Hz); δ_C_ 154.1, 152.9, 128.8, 118.1, 108.3, 108.2], two methines (one of which is oxygenated) (δ_H_/δ_C_ 4.13/67.1; 4.36/28.9), which were similar to those of (−)-catechin [13], suggesting the presence of flavan-3-ol moiety. HMBC correlations (Figure S5) from H-4 to C-8″, C-9″, and C-10″ suggested the connection of C-4 to C-9″. The above NMR spectroscopic data of **1** revealed nearly identical structural features to those in lysidiside W [9], except that the C-2 methine was replaced by one ketal carbon (δ_C_ 109.1 in **1**), and the chemical shift of C-3 (δ_C_ 72.9 in lysidiside W) was slightly upfield (δ_C_ 67.1 in **1**). Considering the chemical shifts of C2/C3 and the fact that **1** has one fewer degrees of unsaturation than lysidiside W, an epoxy ring was assigned at C-2 and C-3, which was further confirmed by HMBC correlations from H-4 to C-2 and C-3, and H-2′/H-6′ to C-2. As a result, the planar structure of **1** was established as shown.

**Figure 2:**
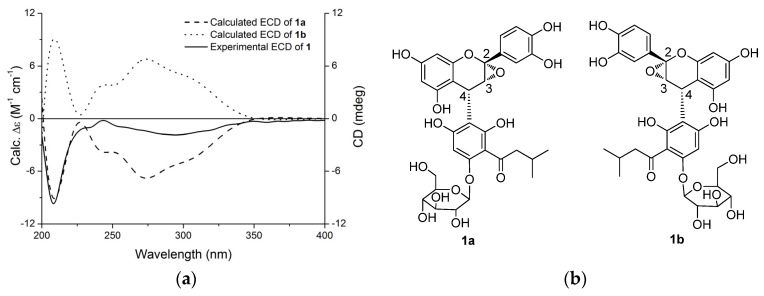
(**a**) Experimental circular dichroism (CD) spectrum of **1** and theoretical calculated electronic CD (ECD) spectra of **1a** and **1b** in MeOH; (**b**) Structures of **1a** and **1b**.

**Figure 3:**
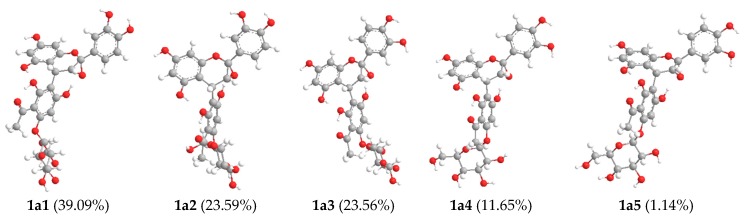
Optimized geometries of predominant conformers and Boltzmann distribution in MeOH of **1a**.

The relative configuration of **1** was determined by analysis of its ^1^H-^1^H coupling constants. A coupling constant of 3.6 Hz between H-3 and H-4 suggested their *cis* relationship [14,15,16,17]. The absolute configuration of C-4 in **1** was deduced based on the experimental and theoretically-calculated circular dichroism (CD) spectra. The experimental CD spectrum of **1** showed a negative Cotton effect at the low wavelength (220–240 nm) (Figure 2) similar to that of lysidiside W [9], suggesting the 4*R*-configuration. Therefore, the absolute configuration of **1** was proposed as 2*S*,3*R*,4*R*, which was further confirmed through electronic circular dichroism (ECD) calculations by time-dependent density functional theory (TD-DFT) [18,19,20,21,22]. According to the established relative configuration, one of the two enantiomers—**1a** or **1b** (Figure 2)—should represent the absolute configuration of **1**. A systematic conformational analysis was performed for **1a** or **1b** with the Molecular Operating Environment (MOE) software package in the MMFF94 molecular mechanics force field. The MMFF94 conformational search provided 10 conformers in an energy window of 5 kcal/mol, which were geometrically optimized at the B3LYP/6-31G(d) basis set level. Five predominant conformers above 1.00% populations occupied a Boltzmann distribution of 39.09%, 23.59%, 23.56%, 11.65%, and 1.14%, respectively (Figure 3). The overall calculated ECD spectra of **1a** and **1b** were then generated by Boltzmann-weighting of these five lowest energy conformers. The experimental ECD spectrum of **1** was nearly identical to the calculated ECD spectrum of **1a** (Figure 2), further confirming the deduction of 2*S*,3*R*,4*R* absolute configuration for **1**.

The β-configuration for the anomeric carbon of glucose was suggested by the large coupling constant (7.6 Hz) of H-1′′′ (δ 4.96) [23], and d-form configuration was determined by GC analysis of the product from enzymatic hydrolysis of **1** [24].

Lysidiside Y (**2**) was obtained as white powder. The pseudomolecular ion [M + Na]^+^ peak at *m*/*z* = 547.1440 (calcd. for C_24_H_28_O_13_Na, 547.1428) by HRESIMS suggested a molecular formula of C_24_H_28_O_13_ with 11 degrees of unsaturation. Analysis of its ^1^H- and ^13^C-NMR spectroscopic data (Table 1) revealed the presence of the identical isovalerylphloroglucinol glucoside moiety as found in **1** (Figures S7 and S8). The remaining NMR resonances (Figures S7 and S8) of **2** were observed as a singlet for two aromatic protons (δ_H_ 7.03), one carbonyl carbon (δ_C_ 168.3), and six aromatic carbons on symmetrical substituted benzene ring [δ_C_ 146.5 (2C), 139.9, 121.3, 110.3 (2C)], suggesting the presence of a galloyl moiety [11]. HMBC (Figure S10) correlation from H-6′ to C-1″ located the galloyl moiety at C-6′. Therefore, the planar structure of **2** was established as depicted. The relative configuration (β) for the glucose in **2** were determined on the basis of the coupling constant (7.5 Hz) of the anomeric proton, and d-configuration was identified by comparison with authentic d-glucose using GC analysis, similarly to the method of configuration determination for the glucose of **1**.

The known compounds **3** and **4** were identified as 2-(2-methylbutyryl)phloroglucinol 1-*O*-β-d-glucopyranoside and (*E*)-resveratrol 3-(6″-galloyl)-*O*-β-d glucopyranoside, respectively, by extensive analysis of their ESIMS, ^1^H- and ^13^C-NMR data, as well as comparison with those reported [10,11].

### 2.3. Antioxidative Activity of Compounds ***1*** and ***2***

Compounds **1** and **2** were tested for antioxidative activity, and vitamin E (type VI) was used as positive control. Both **1** and **2** showed significant antioxidant activity, with IC_50_ values of 12.0 and 11.8 µM, respectively, while vitamin E showed an IC_50_ value of 33.4 µM.

## 3. Materials and Methods

### 3.1. General Experimental Procedures

Optical rotations were determined on a Perkin-Elmer 241 automatic digital polarimeter (Perkinelmer, Waltham, MA, USA). CD spectrum was obtained from a Jasco-815 CD spectrometer (Tokyo, Japan). IR spectra were recorded on a Nicolet 5700 FT-IR spectrometer (Madison, SD, USA). 1D-and 2D NMR spectra were recorded on INOVA-500 and MP-400 spectrometers (Varian, Palo Alto, CA, USA) with TMS as internal standard. HRESIMS spectra were recorded on an Autospec-Ultima ETOF Spec mass spectrometer (Waters, Milford, DE, USA).

### 3.2. Computational Details

Systematic conformational analyses for **1** were performed via the Molecular Operating Environment (MOE) version 2009.10 [25] software package using the MMFF94 molecular mechanics force field calculation. The MMFF94 conformational analyses were further optimized using TD-DFT at the B3LYP/6-31G(d) basis set level. The stationary points have been checked as the true minima of the potential energy surface by verifying that they do not exhibit vibrational imaginary frequencies. The 10 lowest electronic transitions were calculated, and the rotational strengths of each electronic excitation were given using both dipole length and dipole velocity representations. ECD spectra were simulated using a Gaussian function with half-bandwidths of 0.35 eV. Equilibrium populations of conformers at 298.15 K were calculated from their relative free energies (ΔG) using Boltzmann statistics. The overall ECD spectra were then generated according to Boltzmann weighting of each conformer. The systematic errors in the prediction of the wavelength and excited-state energies are compensated for by employing UV correlations. All quantum computations were performed using the Gaussian09 package [26].

### 3.3. Plant Materials

The roots of *L. rhodostegia* were collected from Guangxi Province of China in December 2006. The sample was identified by Professor Shou-Yang Liu (GuangXi College of Traditional Chinese Medicine, Nanning, China), and a voucher specimen (No. 002775) has been deposited in the Herbarium of Institute of Materia Medica, Chinese Academy of Medical Sciences, Beijing, China.

### 3.4. Isolation and Purification of Compounds ***1***–***4***

The air-dried roots of *L. rhodostegia* (4.7 kg) were extracted with 95% EtOH (10 L × 3) and concentrated in vacuo to give the crude extract (563 g), which was suspended in water, and then successively extracted with *n*-hexane, EtOAc, and *n*-BuOH. The EtOAc extract (156 g) was chromatographed on ODS eluting with MeOH–H_2_O (30:70–85:15), and then purified by Sephadex LH-20 (MeOH:H_2_O, 1:1). The resulting subfractions were combined and further purified by preparative RP-HPLC (YMC-Pack ODS-A column, 10 µm, 20 mm × 250 mm, 30% MeOH in H_2_O, 5 mL/min) to afford **1** (17 mg, *t*_R_ = 17.5 min), **2** (35 mg, *t*_R_ = 22.3 min), **3** (10 mg, *t*_R_ = 20.3 min), and **4** (5 mg, *t*_R_ = 25.3 min), respectively.

### 3.5. Characterization of Compounds ***1***–***4***

*Lysidiside X* (**1**). Pale yellow powder, ${\left[\alpha \right]}_{D}^{20}$ +45.2 (*c* 0.1, MeOH); UV λ_max_ 210 nm; CD (MeOH): λ (Δε) 208 (−10.0), 243 (−0.2), 293 (−1.9), 360 (−0.4); IR (KBr) *v*_max_ 3378, 2956, 1618, 1501, 1436, 1120, 1075 cm^−1^ (Figure S1); NMR data see Table 1; HRESIMS: *m*/*z* 681.1763 [M + Na]^+^ (calcd. for C_32_H_34_O_15_Na, 681.1795).

*Lysidiside Y* (**2**). White powder, ${\left[\alpha \right]}_{D}^{20}$ ‒46.8 (*c* 0.1, MeOH); UV λ_max_ 210 nm; IR (KBr) *v*_max_ 3456, 2959, 1704, 1634, 1602, 1454, 1174, 1080 cm^−1^ (Figure S6); NMR data see Table 1; HRESIMS: *m*/*z* 547.1440 [M + Na]^+^ (calcd. for C_24_H_28_O_13_Na, 547.1428).

*2-(2-Methylbutyryl)phloroglucinol 1-O-*β*-d-glucopyranoside* (**3**). White powder, ${\left[\alpha \right]}_{D}^{20}$ ‒35.9 (*c* 0.1, MeOH); UV λ_max_ 210, 220, 280 nm; IR (KBr) *v*_max_ 3356, 2936, 1637, 1605, 1545, 1450, 1173, 1088 cm^−1^; ^1^H-NMR (CD_3_OD, 500 MHz): δ_H_ 6.06 (1H, d, *J* = 2.0 Hz), 5.83 (1H, d, *J* = 2.0 Hz), 4.99 (1H, d, *J* = 7.5 Hz), 3.85 (1H, dd, *J* = 12.0, 1.5 Hz), 3.70 (1H, m), 3.66 (1H, dd, *J* = 12.0, 5.0 Hz), 3.38 (1H, m), 3.45 (1H, m), 3.28 (1H, m), 3.24 (1H, m), 1.60 (1H, m), 1.26 (1H, m), 0.95 (3H, t, *J* = 6.0 Hz), 0.84 (3H, t, *J* = 7.0 Hz); ^13^C-NMR (CD_3_OD, 125 MHz): δ_C_ 207.2, 167.7, 166.9, 162.3, 105.8, 101.8, 98.2, 96.6, 78.6, 78.3, 74.9, 71.2, 62.4, 46.8, 29.2, 16.8, 12.0; ESIMS: *m*/*z* 373.1 [M + H]^+^.

*(E)-Resveratrol 3-(6″-galloyl)-O-*β*-d-glucopyranoside* (**4**). White powder, ${\left[\alpha \right]}_{D}^{20}$ ‒78.3 (*c* 0.1, MeOH); UV λ_max_ 210, 320 nm; IR (KBr) *v*_max_ 3366, 2936, 1693, 1604, 1511, 1450, 1334, 1068 cm^−1^; ^1^H-NMR (CD_3_OD, 500 MHz): δ_H_ 7.35 (2H, br d, *J* = 8.5 Hz), 6.96 (1H, d, *J* = 16.5 Hz), 6.96 (2H, s), 6.82 (1H, d, *J* = 16.5 Hz), 6.74 (2H, br d, *J* = 8.5 Hz), 6.62 (1H, br s), 6.58 (1H, br s), 6.34 (1H, br s), 4.89 (1H, d, *J* = 7.5 Hz), 4.39 (1H, br d, *J* = 12.0 Hz), 4.33 (1H, dd, *J* = 12.0, 3.0 Hz), 3.67 (1H, m), 3.41 (1H, m), 3.32 (1H, m), 3.26 (1H, m); ^13^C-NMR (CD_3_OD, 125 MHz): δ_C_ 165.8, 158.7, 158.5, 157.3, 145.6 (2C), 139.5, 138.6, 128.6, 128.0 (3C), 125.2, 119.3, 115.5 (2C), 108.6 (2C), 106.8, 105.1, 102.7, 100.6, 76.3, 73.7, 73.3, 69.2, 63.0; ESIMS: *m*/*z* 541.1 [M − H]^−^.

### 3.6. Hydrolysis and Determination of Absolute Configuration of Sugars

A sample of **1** (13 mg) in 2 mL of water was incubated with helicase (26 mg) at 37 °C for 12 h, before being extracted with EtOAc to remove the aglycone. After evaporation of the aqueous layer, 2.0 mg of L-cysteine methyl ester hydrochloride and 100 μL of pyridine were added, and the mixture was stirred at 60 °C for 2 h. Then, 0.2 mL of N-trimethylsilylimidazole was added, and the resulted solution was stirred for another 2 h. The reaction was quenched with 3.0 mL of H_2_O and extracted with *n*-hexane (3 mL × 3), and the organic layer was analyzed by GC. The GC analysis conditions were as follows: capillary column, DB-5 (30 cm × 0.25 mm); detector, FID; detector temperature, 280 °C; injection temperature, 250 °C; initial temperature was maintained at 100 °C for 2 min and then raised to 280 °C at 10 °C/min, and final temperature was maintained for 5 min; carrier, N_2_ gas. The resulting glucose derivative coeluted with a derivatized d-glucose standard (*t*_R_ 23.9 min). A sample of compound 2 (30 mg) in 6 mL of water was treated with tannase (50 mg) at room temperature for 3 h. The reaction mixture was filtered, and the filtrate was concentrated to dryness. The residue was then subjected to Sephadex LH-20 column chromatography using MeOH as eluent to afford gallic acid and a hydrolysate, which was used for derivatization and GC analysis in a similar way as **1**. As a result, the sugar unit in **2** was also determined as d-glucose.

### 3.7. Biological Activity Assessment of Compounds ***1*** and ***2***

The antioxidant activities of **1** and **2** were determined by the content of malondialhyde (MDA), which was produced during microsomal lipid per-oxidation induced by ferrous-cysteine. MDA was detected by using the thiobarbituric acid (TBA) method. Microsomal protein (1 mg/mL), different concentration of compounds, and cysteine (0.2 mM in 0.1 M PBS) were incubated for 15 min at 37 °C. Ferrous (0.5 mM) was added, and the mixture was incubated for another 15 min at the same temperature. An equal volume of 20% TCA was added to terminate the reaction. The above solvent was centrifuged for 10 min at 3000 rpm. The supernatants reacted with 0.67% TBA for 10 min at 100 °C. After being cooled to room temperature, the MDA was determined by the absorbance at 532 nm, and then the inhibitory rates were calculated [27].

## 4. Conclusions

We identified two new potent antioxidative agents—lysidisides X and Y (**1** and **2**)—from the roots of *L. rhodostegia*. Compound **1** is structurally related to lysidiside W [9], but differs from the known compound by the presence of an additional epoxy ring at C-2 and C-3, and represents the first described 4-arylflanvan-3-ol incorporating oxirane, while compound **2** is a new member of the phloroglucinols characterized by galloylated product of lysidiside A [2]. These further demonstrate the genus *Lysidice* as a productive producer of bioactive natural compounds.

## Figures and Tables

**Table 1:** ^1^H-, ^13^C-NMR, and HMBC data for compounds **1** and **2** in CD_3_OD.

No.	Lysidiside X (1)			Lysidiside Y (2)		
	δ_H_ mult. (*J* in Hz) ^a^	δ_C_^b^	HMBC	δ_H_ mult. (*J* in Hz) ^c^	δ_C_^d^	HMBC
1				0.84 d (6.5)	23.3	2, 3, 4
2		109.1		2.15 m	26.3	1, 3, 4, 11
3	4.13 d (3.6)	67.1	10	3.07 dd (15.5, 6.5)	54.1	1, 2, 4, 5, 11
				2.81 dd (15.5, 7.5)		
4	4.36 d (3.6)	28.9	2, 3, 5, 9, 10, 8″, 9″, 10″		207.3	
5		156.8			107.3	
6	5.94 d (2.0)	98.4	5, 7, 8, 10		162.1	
7		158.4		6.11 d (2.0)	95.8	4, 5, 6, 8, 9
8	6.03 d (2.0)	96.6	6, 7, 9, 10		165.7	
9		154.1		5.91 d (2.0)	98.6	5, 7, 8, 10
10		103.7			167.5	
11				0.83 d (6.5)	22.8	2, 3, 4
1′		128.8		5.03 d (7.5)	102.2	6, 2′, 3′, 5′
2′	6.88 d (2.0)	108.3	2, 1′, 3′, 4′, 6′	3.67	74.8	
3′		152.9		3.49	78.3	
4′		154.1		3.52	71.2	
5′	6.56 d (8.0)	108.2	1′, 3′	3.49	75.8	
6′	6.91 dd (8.0, 2.0)	118.1	2, 2′, 4′, 5′	4.56 dd (12.0, 1.0)	64.2	4′, 5′, 1″
				4.35 dd (12.0, 5.0)		
1″	0.89 d (6.8)	22.9	2″, 3″		168.3	
2″	2.21 m	26.1	3″		121.3	
3″	3.14 (16.0, 6.4)	54.3	1″, 2″, 4″, 11″	7.03 s	110.3	1″, 2″, 4″(6″), 5″
	2.88 dd (16.0, 7.2)					
4″		208.2			146.5	
5″		108.2			139.9	
6″		160.2			146.5	
7″	6.33 s	96.2	5″, 6″, 8″, 9″	7.03 s	110.3	1″, 2″, 4″(6″), 5″
8″		159.6				
9″		110.4				
10″		161.2				
11″	0.92 d (6.8)	23.3	1″, 2″, 3″			
1′′′	4.96 d (7.6)	102.1	6″, 2′′′, 3′′′, 5′′′			
2′′′	3.45	74.9				
3′′′	3.39	78.3				
4′′′	3.35	71.2				
5′′′	3.39	78.4				
6′′′	3.84 br d (12.0)	62.3				
	3.65 dd (12.0, 5.0)					

^a^ Recorded at 400 MHz; ^b^ Recorded at 100 MHz; ^c^ Recorded at 500 MHz; ^d^ Recorded at 125 MHz.

## Supplementary Materials

Supplementary materials are available online.
