# Supplementary material for: Phloroglucinols with Antioxidant Activities Isolated from Lysidice rhodostegia
Source: Molecules. 2017 May 23;22(6):855. doi: 10.3390/molecules22060855 (PMC6152794; doi:10.3390/molecules22060855)

Supplementary Matrials: Phloroglucinols with Antioxidative activity from the Roots of *Lysidice rhodostegia*

Xian-Fu Wu [1,2,] *, Li Li [1], Yong Li [1], Hai-Ning Lv [1], Yun-Bao Liu [1], and You-Cai Hu [1,]*

[1] State Key Laboratory of Bioactive Substance and Function of Natural Medicines, Institute of Materia Medica, Chinese Academy of Medical Sciences and Peking Union Medical College, Beijing 100050, China; annaleelin@imm.ac.cn (L.L.); liyong@imm.ac.cn (Y.L.); lhn371102@126.com (H.-N.L.); liuyunbao@imm.ac.cn (Y.-B.L.)

[2] National Institutes for Food and Drug Control, Beijing 100050, Beijing, China

* Correspondence: huyoucai@imm.ac.cn (Y.-C.H); wuxf99@163.com (X.-F.W.); Tel.: +86-10-6127-1883 (Y.-C.H); Tel.: +86-10-5385-2025 (X.-F.W.)

Contents .... **Page**

Figure S1. IR spectrum of compound **1** 2

Figure S2. ^{1}H-NMR spectrum of compound **1** (400 MHz, CD_3OD) 3

Figure S3. ^{13}C-NMR spectrum of compound **1** (100 MHz, CD_3OD) 4

Figure S4. HMQC spectrum of compound **1** 5

Figure S5. HMBC spectrum of compound **1** 6

Figure S6. IR spectrum of compound **2** 7

Figure S7. ^{1}H-NMR spectrum of compound **2** (500 MHz, CD_3OD) 8

Figure S8. ^{13}C-NMR spectrum of compound **2** (125 MHz, CD_3OD) 9

Figure S9. HMQC spectrum of compound **2** 10

Figure S10. HMBC spectrum of compound **2** 11

Figure S1. IR spectrum of compound **1**.

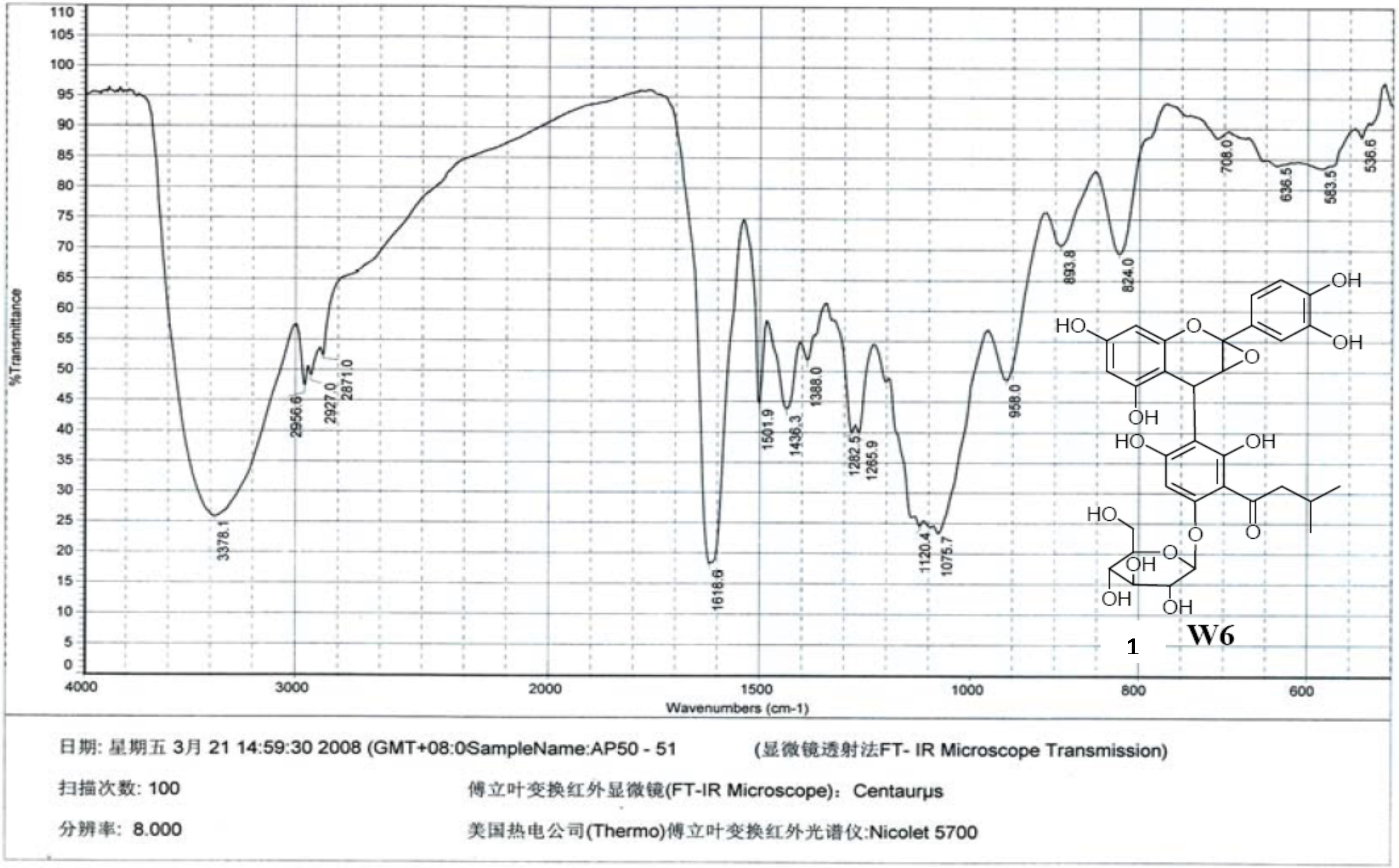

Note:

日期：星期五 3 月 21 (Date: Friday Mar. 21)

显微镜透射法 (Microscope Transmission Method)

扫描次数(Scan times)

分辨率(Resolution)

傅立叶变换红外光谱仪(Fourier Transform Infrared Spectrometer)

Figure S2. ^{1}H-NMR spectrum of compound **1** (400 MHz, CD_3OD).

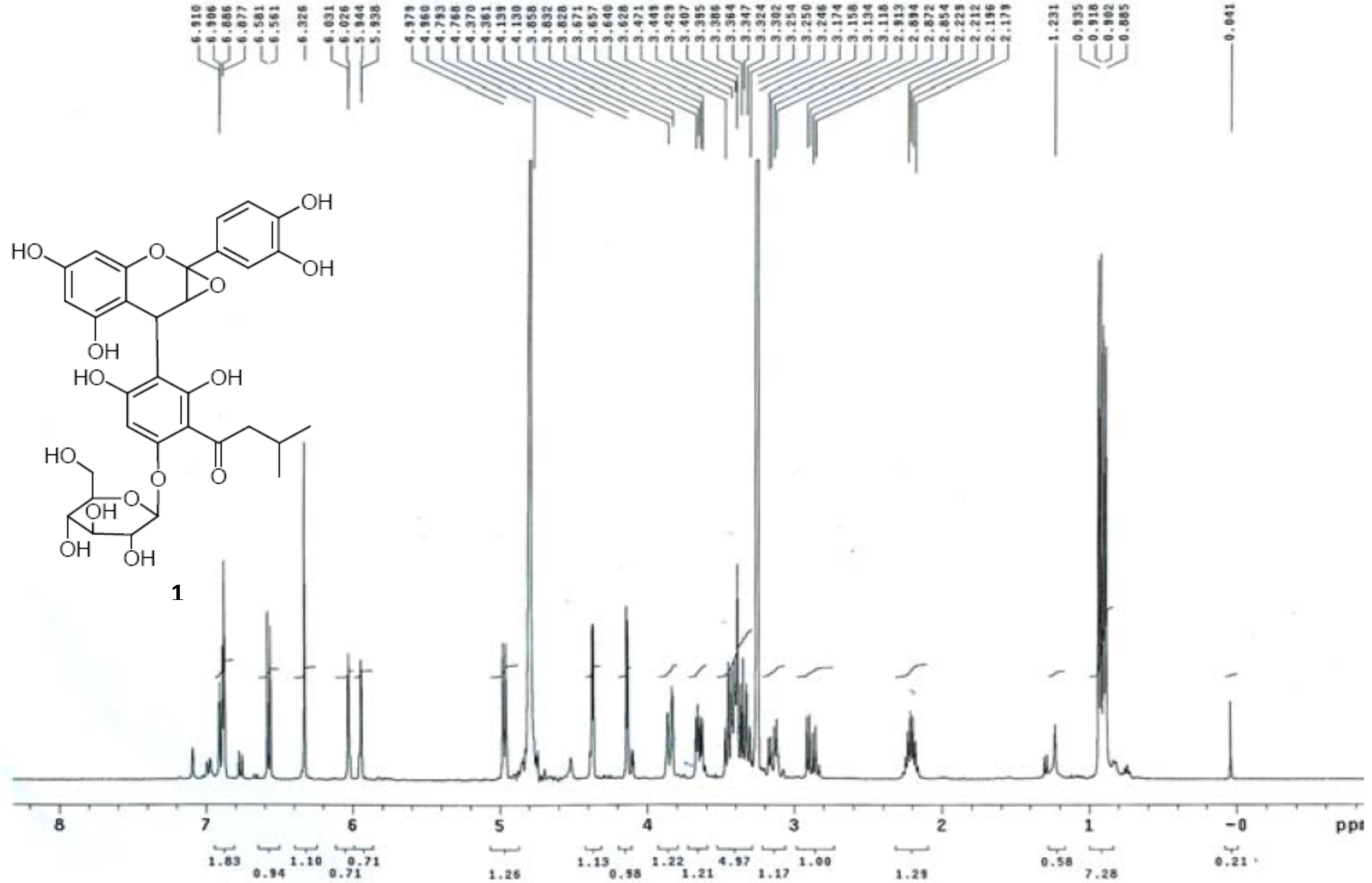

Figure S3. ^{13}C-NMR spectrum of compound **1** (100 MHz, CD_3OD).

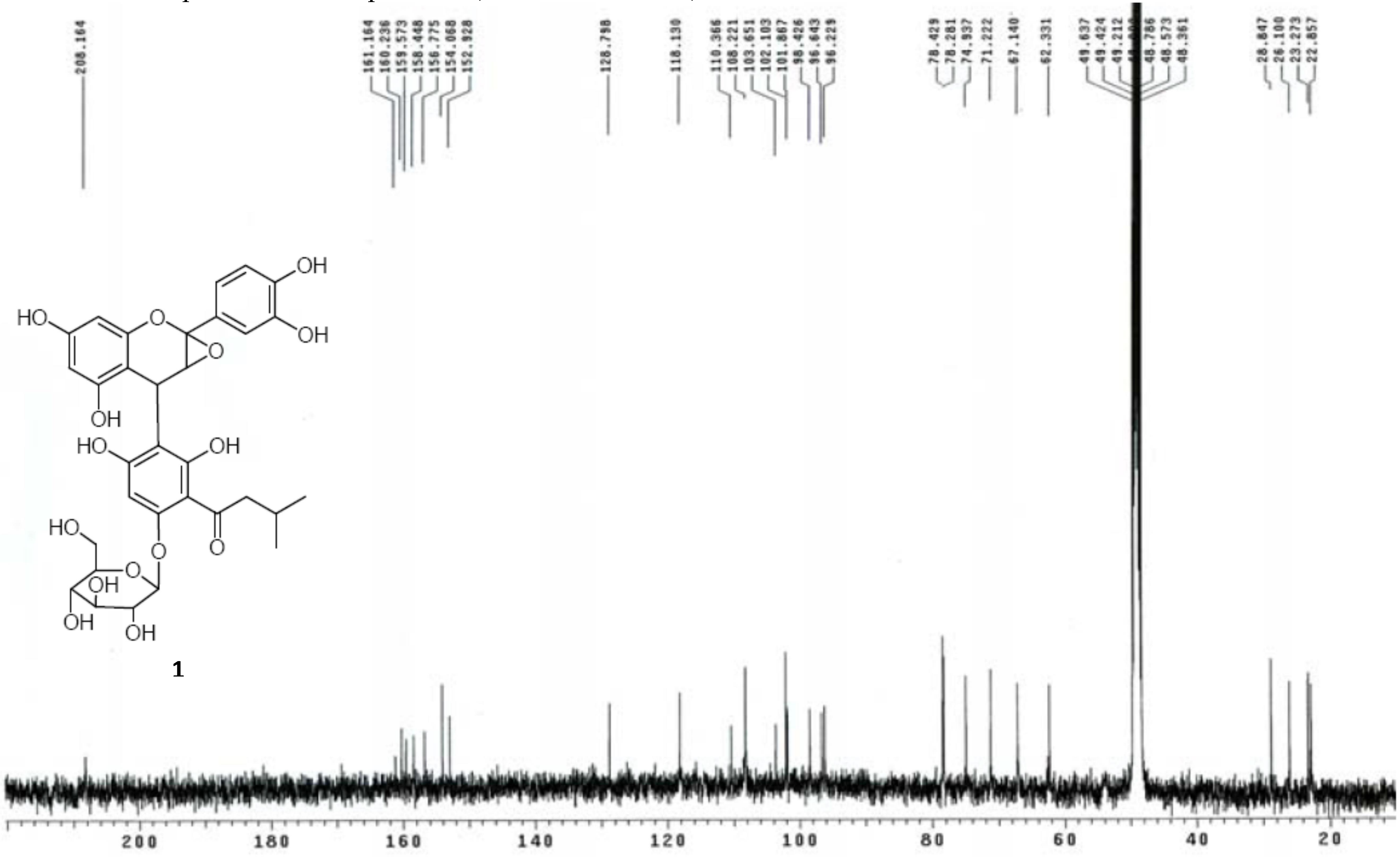

Figure S4. HMQC spectrum of compound **1**.

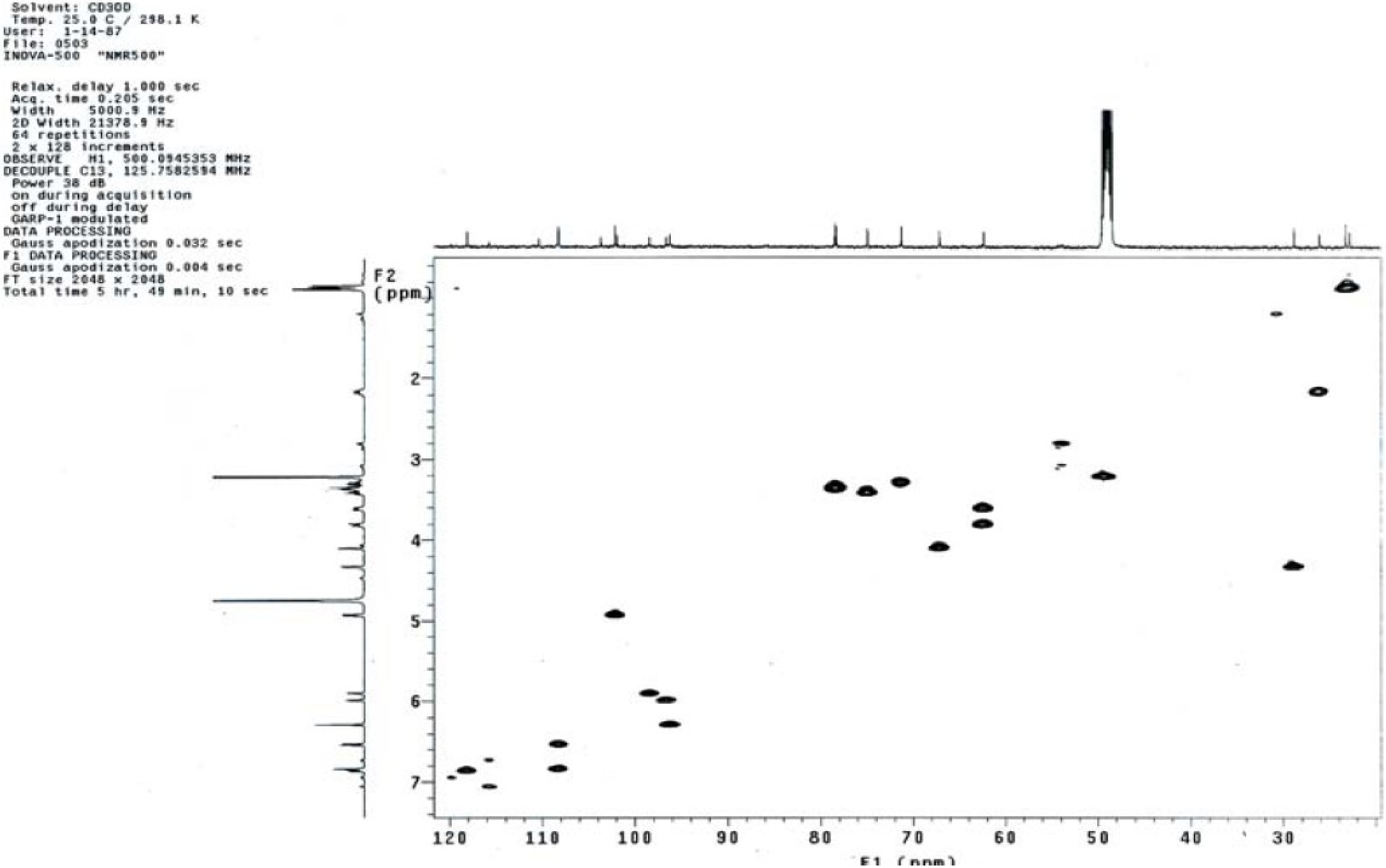

Figure S5. HMBC spectrum of compound **1**.

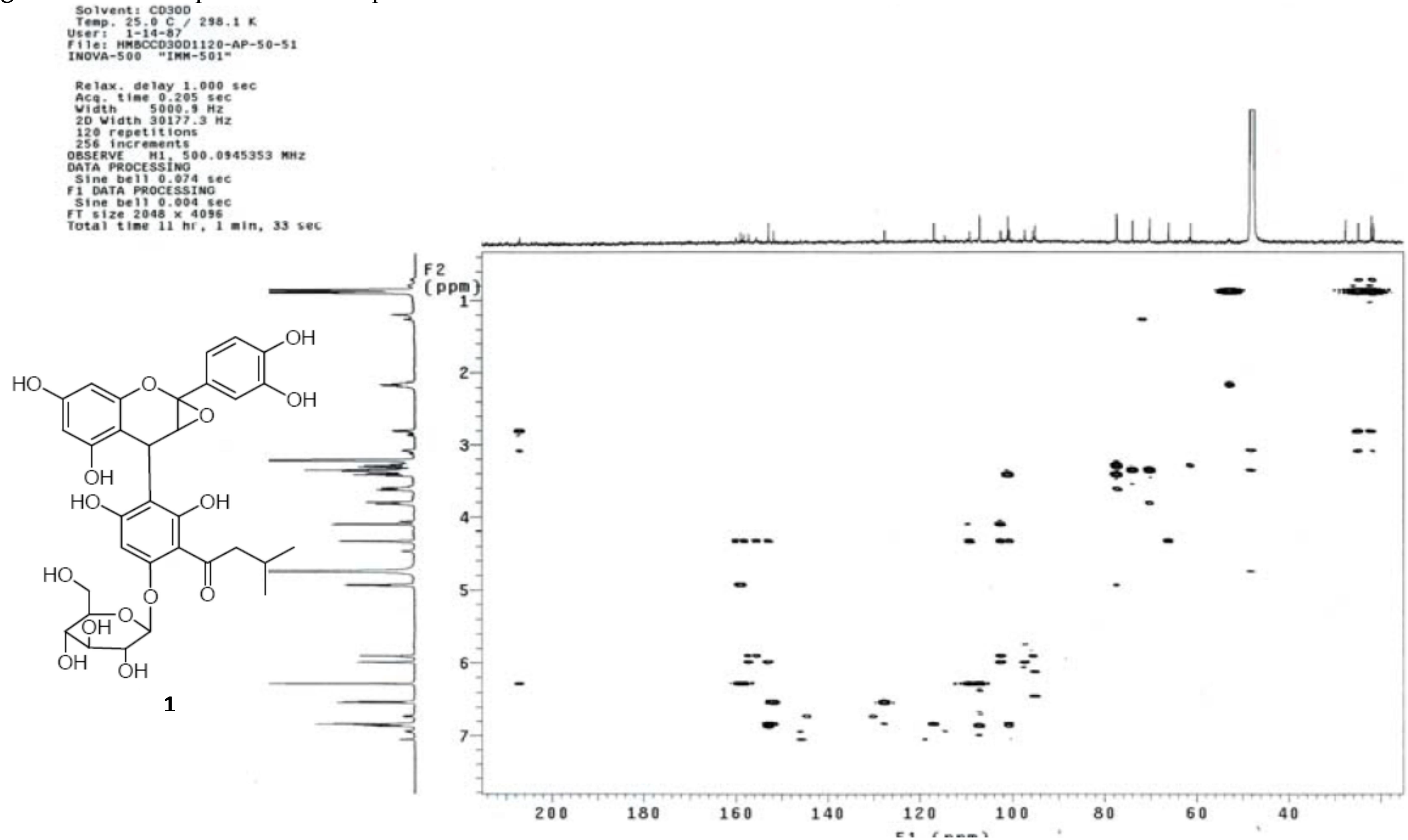

Figure S6. IR spectrum of compound **2**.

Note:

日期：星期五 3 月 21 (Date: Friday Mar. 21)

显 微 镜 透 射 法 (Microscope Transmission Method)

扫描次数(Scan times)

分辨率(Resolution)

傅立叶变换红外光谱仪(Fourier Transform Infrared Spectrometer)

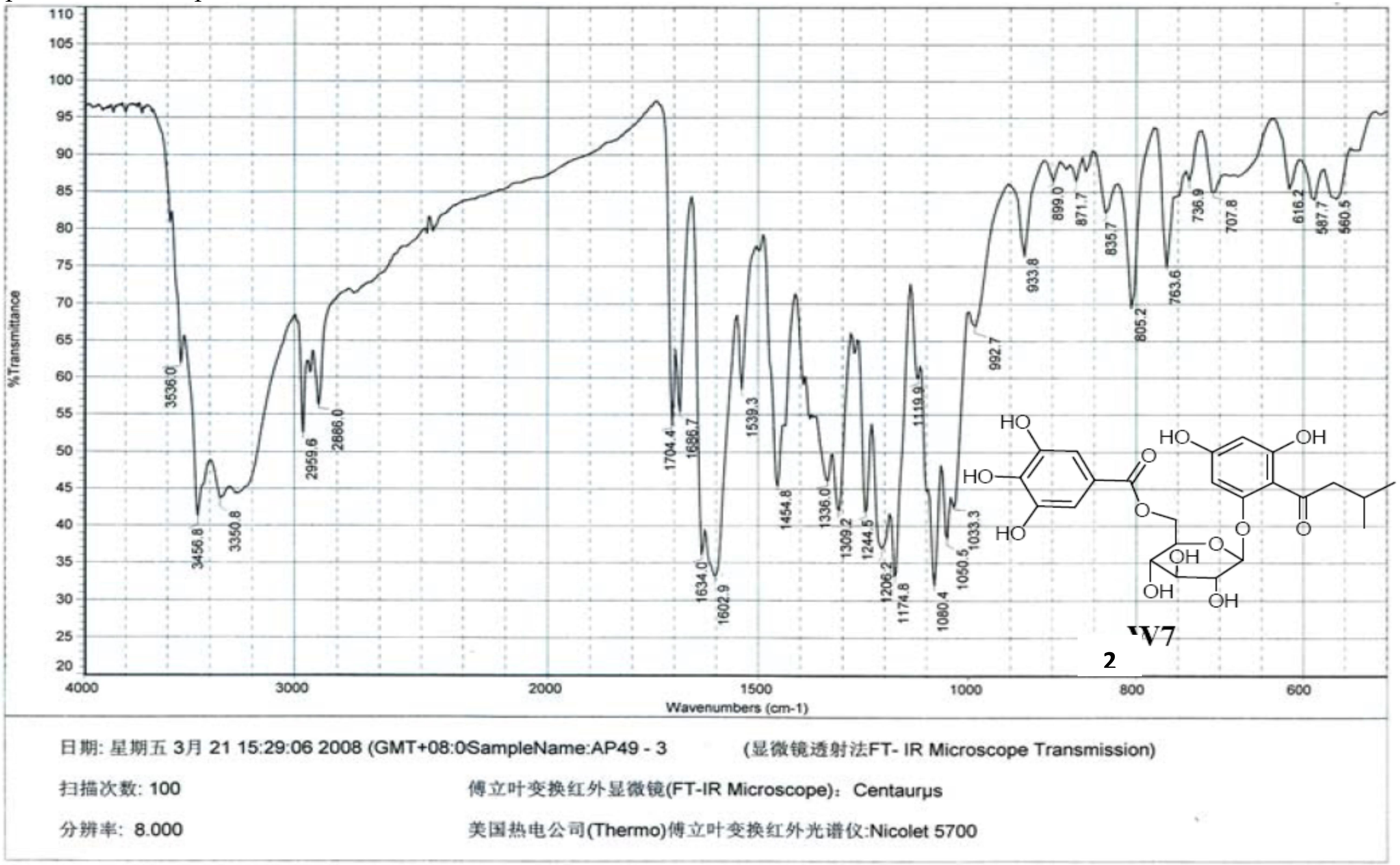

Figure S7. ^{1}H-NMR spectrum of compound **2** (500 MHz, CD_3OD).

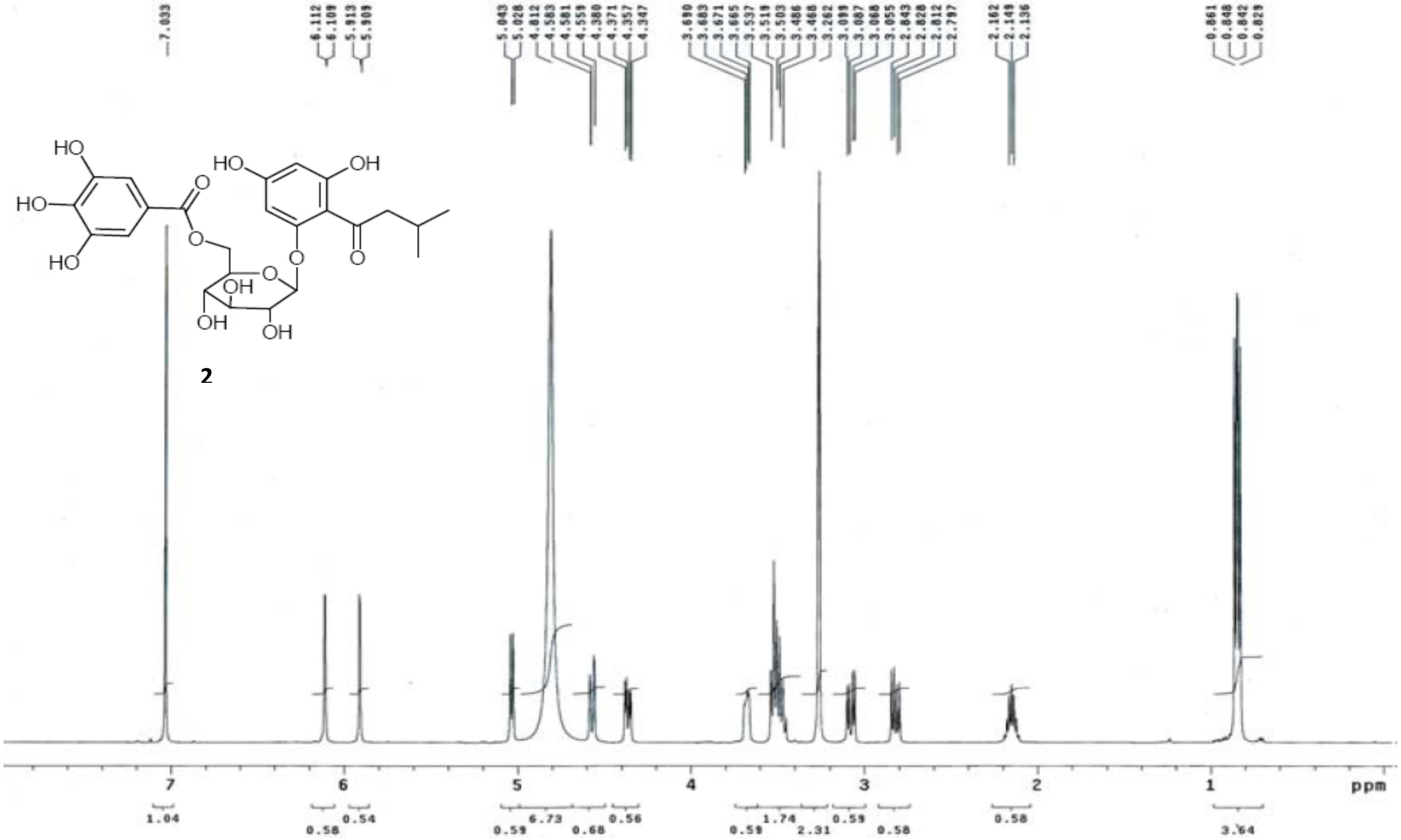

Figure S8. ^{13}C-NMR spectrum of compound **2** (125 MHz, CD_3OD).

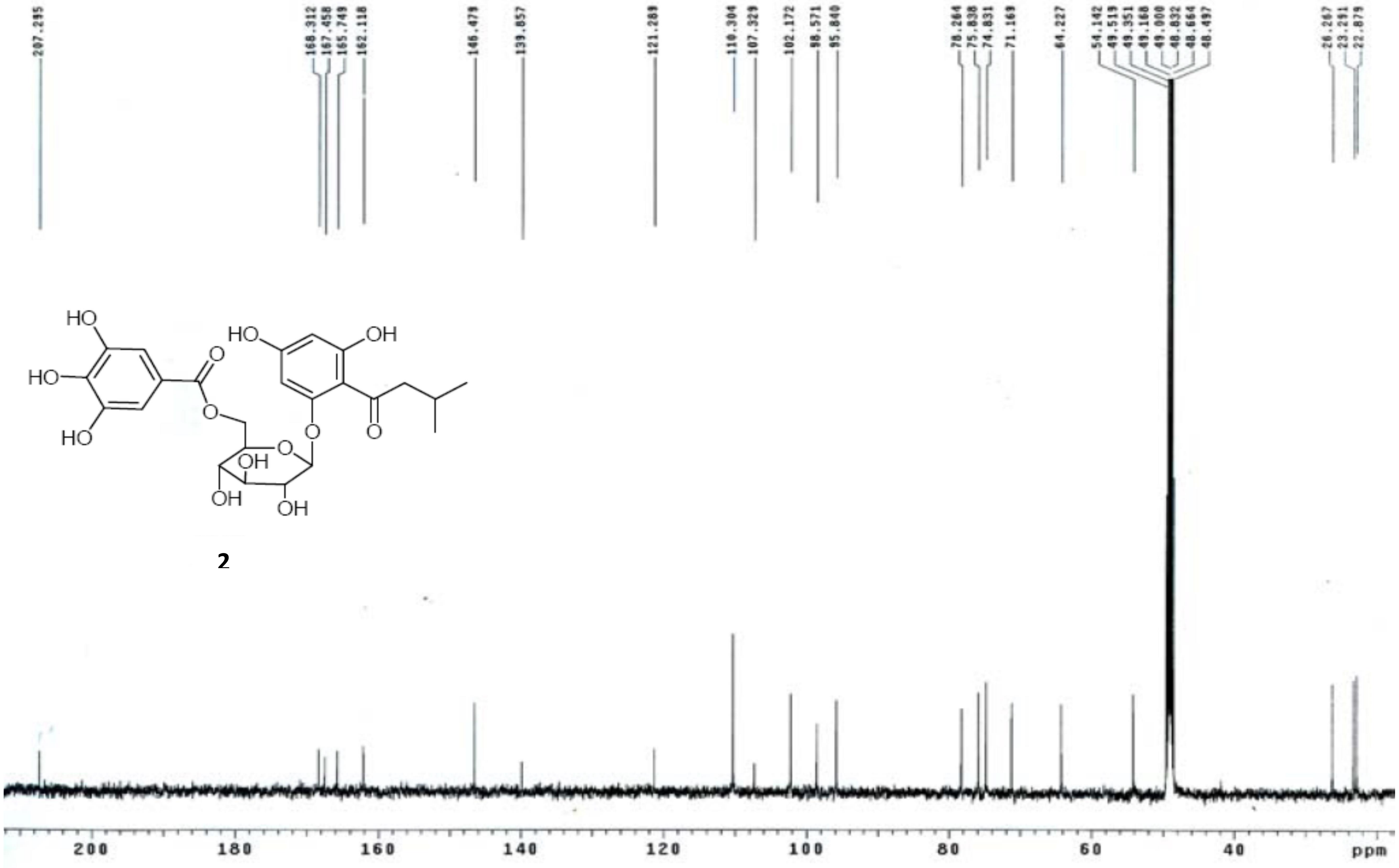

Figure S9. HMQC spectrum of compound **2**.

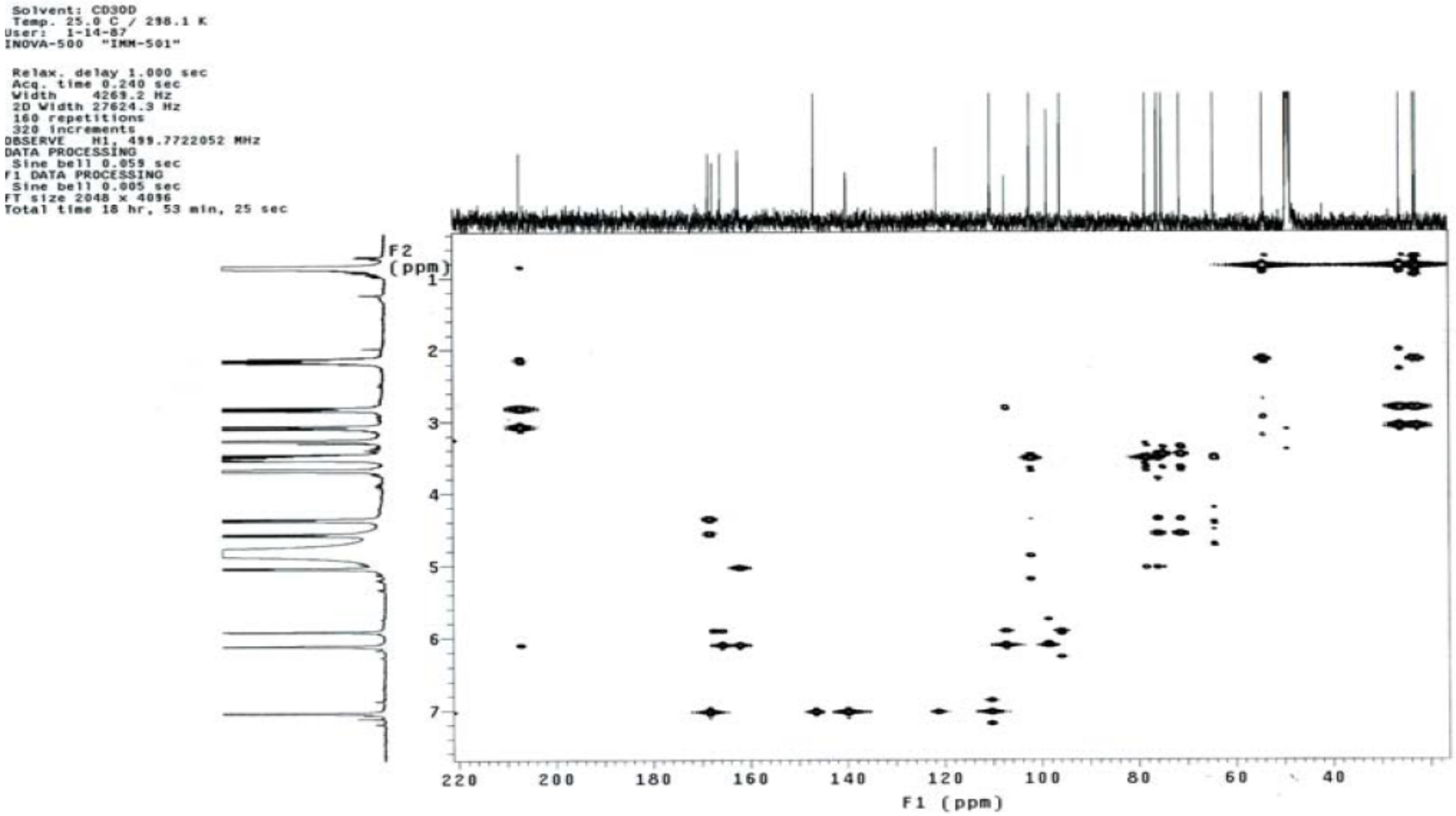

Figure S10. HMBC spectrum of compound **2**.

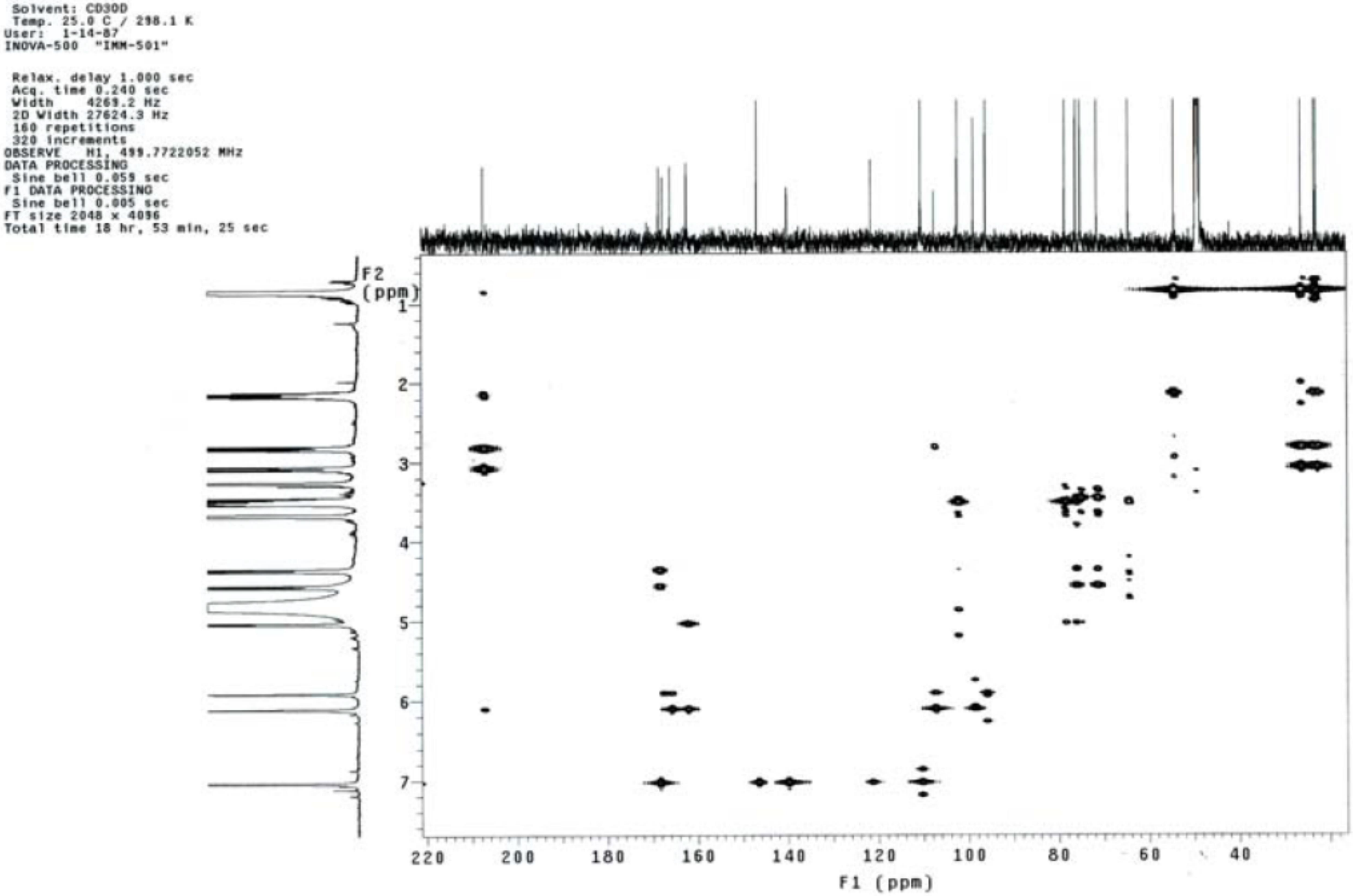

Supplement: Supplementary file 1 [file molecules-22-00855-s001.pdf]
